# Satisfaction of Nursing Care and Its Associated Factors Among Patients With Coronary Artery Disease at Jakaya Kikwete Cardiac Institute, Dar es Salaam, Tanzania: A Cross‐Sectional Study

**DOI:** 10.1155/nrp/9954455

**Published:** 2025-12-06

**Authors:** Theresia I. Marombe, Masunga K. Iseselo, Samweli Kisakeni

**Affiliations:** ^1^ Directorate of Clinical Nursing, Jakaya Kikwete Cardiac Institute, Dar es Salaam, Tanzania; ^2^ Department of Clinical Nursing, Muhimbili University of Health and Allied Sciences, Dar es Salaam, Tanzania, muchs.ac.tz

**Keywords:** coronary artery disease, factors, nursing care, satisfaction

## Abstract

**Background:**

Patient satisfaction is a measure of the effectiveness of care, particularly in patients with coronary artery disease (CAD). It has been demonstrated that one of the factors affecting a patient’s quality of life is their satisfaction with the nursing care they receive. Inadequate nursing care can have negative effects on patients’ satisfaction and, if appropriate action is not taken, may deter patients from visiting hospitals. This study aimed to assess the level of satisfaction with nursing care and its associated factors among patients with CAD.

**Methods:**

A cross‐sectional hospital‐based study was conducted at the Jakaya Kikwete Cardiac Institute (JKCI) in Dar es Salaam, Tanzania. A total of 150 respondents were recruited using a simple random sampling technique. Data were collected using a standardised questionnaire. Univariate and multivariate logistic regression analyses were performed to assess the association of sociodemographics, level of patient satisfaction and factors associated with patient satisfaction with nursing care. A *p* value of < 0.05 was considered statistically significant.

**Results:**

The mean age of respondents was 58.78 ± 15.16 years, with the majority, 81 (54%), being males. Most 106 (70.7%) respondents were married, and 103 (68.7%) were peasants. Most respondents, 127 (85%), were satisfied with nursing care. Patients with higher education and treatment category with VIP services had significantly reduced chances of satisfaction with nursing care (adOR 0.478, 95% CI: 0.094–0.632, *p* = 0.012) and adOR 0.234 of 95% CI: 0.074–0.432, *p* = 0.002, respectively. Other factors were not statistically associated with satisfaction with nursing care.

**Conclusion:**

The majority of patients were satisfied with the nursing care. Treatment categories by VIP, insurance and higher education were associated with satisfaction with nursing care. Further research is needed to qualitatively assess the aspects of nursing care that lead to the satisfaction among patients with CAD.

## 1. Introduction

Cardiovascular diseases (CVDs) account for one‐third of all deaths globally, with coronary artery disease (CAD) taking the largest percentage [1]. Studies from Europe and Central Asia reported that CAD contributed to 30% of all deaths in 2005 [2]. In America, the prevalence of CAD has been shown to decrease from 6.2% in 2011 to 6.0% in 2018 [3]. The burden of CAD is greatest in low‐ and middle‐income nations, where it accounts for 129 million DALYS and over 7 million deaths annually [4]. Due to the rising incidence of risk factors such as obesity, hypertension, diabetes, dyslipidaemia and cigarette smoking, the prevalence of CAD is gradually increasing throughout Africa [5]. In this region, CAD is associated with severe disease burden as a result of disparities in diagnosis and management compared to other parts of the world [5]. Hospital mortality in this region was reported to range between 1.2% and 24.5%, with the highest rate reported in Ethiopia [6]. In Tanzania, the prevalence of CAD has been reported to be 22.3% [7], with a mortality rate of 4.5% in urban areas [8].

Apart from pharmacological and interventional management of patients with CAD, social and psychological support is an important part of management, as patients who develop acute coronary syndrome (ACS) are at increased risk of recurrent attacks [9]. Also, patients who undergo revascularisation therapy need psychological support during recovery [9]. This management part involves good patient care during admission and after discharge. During admission, psychological support by doctors and nurses through good and effective communication, timely feedback and counselling on coping skills is mandatory. This has been shown to improve outcomes by helping prevent post‐myocardial infarction (MI), depression and other psychological problems [10]. Organised patient services play a crucial role in understanding, measuring and fulfilling customer desires.

Patient satisfaction is a measure of the effectiveness of the health system and serves as a basis for pressuring medical professionals to take greater responsibility for their patients, which in turn improves survival rates [11]. Within the hospital setting, patient satisfaction encompasses the delicate balance between patient perceptions and their expectations of care [12–14]. Patient satisfaction is expressed in the distribution, availability and use of health services, which is linked to increased treatment adherence and better health outcomes [15]. Patients feel satisfied when they are provided with the necessary care, treatment and a good hospital environment [16]. The process used in the evaluation of patient satisfaction in nursing care includes a cognitive assessment of the services’ structure, method of health delivery and health outcomes [17, 18]. Patients’ emotional reactions are proven to be just as significant as their cognitive assessments of the medical interaction. [19]. A person’s decision to seek medical advice, adhere to treatment and establish a continued relationship with healthcare providers is significantly influenced by their level of satisfaction with the care they get [20].

One of the predictors of good patient satisfaction is good nursing care [16]. Nursing care is an important aspect of improving the quality of life of patients with CAD. Nursing care not only for patients with CAD but also in other diseases is an important part of management, contributing to better health outcomes [16]. These include nurses who efficiently and appropriately give patients information, competent and consistent treatment and tailored patient care delivered with empathy and compassion [19]. Nursing staff pose a significant chance to influence patients’ behaviour and attitudes associated with the whole process of care, rehabilitation and recovery and hence patient satisfaction [16, 19, 21].

To assess this management component and improve its implementation, it is necessary to determine the level of patients’ satisfaction while undergoing treatment for CAD. Despite being an important part of influencing positive patient outcomes, the evaluation of CAD patients’ satisfaction with nursing care has not been well documented, particularly in the Tanzanian context.

Therefore, the purpose of this study was to evaluate the satisfaction of patients with CAD with the nursing care they receive at the Jakaya Kikwete Cardiac Institute (JKCI). The findings of this study are useful in creating strategies for enhancing patient care and improving the overall quality of life, particularly when receiving care.

## 2. Materials and Methods

### 2.1. Design

This was a cross‐sectional hospital‐based study conducted to assess the level of satisfaction with nursing care and identify associated factors among patients with CAD. This design was specifically preferred as we aimed to achieve patient satisfaction and its associated factors relatively faster [22] that could help to provide insight for further planning and nursing care for patients in the study setting.

### 2.2. Setting

The study was carried out in the Jakaya Kikwete Cardiac Institute’s (JKCI) inpatient department (IPD) in Dar es Salaam, Tanzania. Dar es Salaam is the largest commercial city in Tanzania and the home of roughly 5,383,728 people [23]. JKCI is a nationally recognised teaching hospital with a focus on cardiovascular treatment, education and research. Currently, JKCI has 175 nurses, of whom 160 are working as permanent employees, and 15 nurses are working under contract. Nurse–patient ratio differs with the unit. In the ICU, the ratio is 1:1, high dependent unit, 1:4, and in the general ward, it is 1:8 [24]. Patients with CVDs from all over the country are coming to get cardiac services at the institute. The facility has 157 beds and can host 150 inpatient stays and more than 1800 outpatient attends per week [25]. As a result, it was a favourable setting for carrying out this study due to the availability of respondents.

### 2.3. Population

The study involved patients who were admitted with CAD. Both male and female patients were included in this study. The following were the inclusion criteria to participate in this study: inpatients diagnosed with CAD and aged 18 years or above. The patient should have been hospitalised for more than 2 days prior to the day of data collection. However, patients who could not communicate, had memory problems, were seriously ill, had trouble hearing and not willing to participate were excluded from the study because they would not be able to provide accurate information.

### 2.4. Sample Size Estimation and Sampling Techniques

The sample size was calculated using the Krejcie and Morgan formula for a known population as described by Uakaran et al. [26], where *N* = size of the population 200, *X* ^2^ = 3.841 for 95% confidence level and *ε* = margin of error, 5%. Using 50% proportion (P) and the nonresponse rate of 12%, the required sample size of 150 participants was estimated. Simple random sampling was used to select the participants. All patients admitted were listed and assigned consecutive numbers of the same digits from zero to the required sample size. The number was selected from the table of random numbers based on the number given to the respondents. If the number from the random number table corresponds to the number assigned to the respondent, the potential respondent was included in the study. The same technique was repeated until the desired sample size was met.

### 2.5. Data Collection Tools and Procedure

#### 2.5.1. Data Collection Tools

A structured Kiswahili questionnaire translated following the principles proposed by Tsang et al. [27] was used for data collection. During this process, a multistep forward–backward translation of the questionnaire from the English language to Kiswahili and back to the English language was undertaken by two professionals who were experts in both languages. The two professionals independently translated the original questionnaire from the English language to Kiswahili. Following the initial translation, they met to discuss the translation process and produced a single copy of the translated questionnaire by consensus. The tool consisted of demographic data (age, sex, address, level of education, occupation and marital status) and questions related to patient satisfaction with nursing care. The data related to patient satisfaction with nursing care were collected using a Patient Satisfaction with Nursing Care Quality Questionnaire (PSNCQQ), which is a standardised tool [28]. The PSNCQQ consists of 19 items and 4 additional questions related to the assessment of the quality of nursing care. Participants responded by assessing their level of agreement with each statement on a Likert‐type scale from 1 (Excellent) to 5 (Poor). The participants’ responses to the questions were recorded and calculated as the sum of all scale responses. Lower total scores indicate greater satisfaction with nursing care. The responses were categorised as being satisfied when the total score was 19–65, and not satisfied when the score was 66–95. The decision was based on our study context for quality improvement and benchmarking with other studies [18, 29]. None of the items were negatively phrased, and hence reverse response scoring system was not applied.

#### 2.5.2. Pretesting the Tool

Validity in a study refers to how well the results among the study participants represent true findings among similar individuals outside the study [30]. While there was no specific information about the PSNCQQ being directly validated in Tanzania, the instrument had been widely used and validated in other countries with high internal consistency (Cronbach’s (*α*) = 0.98) [31]. To enhance validity in the Tanzanian context, the questionnaire was given to 3 nurses expert in cardiovascular nursing care for review and inputs. Their inputs improved the content of questions in the tool. Also, 30 patients were invited to participate in the pretesting of the questionnaire as proposed by Perneger et al. [32]. The results of pretesting helped to determine the clarity, relevance and accuracy of the questionnaire, as well as the duration a participant would use to respond to the questions. Items which were not clear in the questionnaire were removed, and modifications were made in close consultation with other researchers and clinicians such as expert nurses and doctors in the respective wards. In addition, the Cronbach’s alpha of 0.85 was estimated, which was shown to be acceptable, consistent and relatively free from errors.

#### 2.5.3. Data Collection Procedure

Data collection activity commenced on the 1st to 30th of June 2024. Participants were approached after the ward round to avoid interference with the provision of care. The patients who agreed to participate in the study were provided with an explanation about the purpose of the study, and they signed informed consent forms. Those who refused to participate reported that they did not have the time or were just not interested in participating. We personally administered the questionnaires to all participants. For patients who were not literate, we read out the questionnaire to them and recorded their responses. Participants were allowed to complete the questionnaire at their own convenience. Data collection took place over a period of 4 weeks. The process of filling a single questionnaire took an average of 30 min, and the data collection activity lasted 30 days.

### 2.6. Study Variables

The independent variables were age, sex, education, occupation and treatment category, and they were assessed using a structured questionnaire. The dependent variable was patient satisfaction with nursing care, and it was assessed using PSNCQQ.

### 2.7. Data Management and Analysis

Data cleaning was done by running frequencies to check for any missing variables, which were corrected by checking the questionnaire. Data were analysed using IBM SPSS version 23.0. Continuous data, like age, were categorised and coded before analysis. Descriptive analysis was done for the participants’ sociodemographic and clinical characteristics to determine frequency, percentages and standard deviations. Before analysis, each piece of data was verified to be complete. Categorical variables were summarised by frequency and percentage. Continuous variables were summarised by mean and standard deviation. To analyse the PSNCQQ using percentage distribution, we calculated the percentage of responses that fall into each of the 5 Likert scale categories (Excellent, Very Good, Good, Fair, Poor) for each of the 19 items. This provided a detailed breakdown of patient satisfaction levels across different aspects of nursing care. The additional three global items (overall hospital care, overall nursing care and intention to recommend) were also analysed in the same way to provide a comprehensive overview of patient satisfaction. The outcome variable was categorised into satisfied and not satisfied. The factors associated with satisfaction were assessed using binary logistic regression analysis to remove the confounders. The variables with a *p* value of less than 0.2 were further analysed using multivariate logistic regression, and a *p* value of less than 0.05 was used to ascertain a statistically significant association.

### 2.8. Ethical Approval and Consent to Participate

Ethical approval was obtained from the Institutional Review Board (IRB) at Muhimbili University of Health and Allied Sciences (MUHAS) with Ref. No. MUHAS‐REC‐05‐2024‐2271. The permission to conduct a study was requested from JKCI management through the Directorate of Research, Training, and Consultancy. In this study, participants were informed about the study and were aware that participation was voluntary and free to withdraw from the study at any time. Also, participants were informed that no payments would be given, and their refusal to participate would not affect their right to treatment. All participants approached agreed to participate and signed the consent form. To ensure confidentiality, participants were informed that code numbers would be used instead of their actual names in all documents.

## 3. Results

### 3.1. Sociodemographic Characteristics of the Respondents

The mean age of respondents was 58.78 ± 15.16 years, with a high proportion (75; 50%) aged 45–64 years. Males constituted 81 (54%) of the respondents. Most participants (106; 70.7%) were married, and a significant portion were peasants (103; 68.7%). In terms of education, the proportion of respondents with secondary education was 56 (37.3%). Regarding patients’ method of payment for hospital bills, a majority (99; 66%) were insured (Table 1).

**Table 1:** Sociodemographic characteristics of the respondents (*N* = 150).

Variable	Category	Frequency	Percent (%)
Age (in years)	18–44	25	16.7
	45–64	75	50.0
	65+	50	33.3

Sex	Male	81	54.0
	Female	69	46.0

Marital status	Single	17	11.3
	Widow/widower	14	9.3
	Married	106	70.7
	Divorced/separated	13	8.7

Occupation	Employed	17	11.3
	Peasant	103	68.7
	Business	30	20.0

Education level	Illiterate	7	4.7
	Primary	55	36.7
	Secondary	56	37.3
	Higher education	32	21.3

Patient’s payment method	Out‐of‐pocket	37	24.7
	NHIF	99	66.0
	VIP	14	9.3

Abbreviations: NHIF = National Health Insurance Fund, VIP = very important person.

### 3.2. Satisfaction With Nursing Care Among Patients With CAD

**Figure 1:**
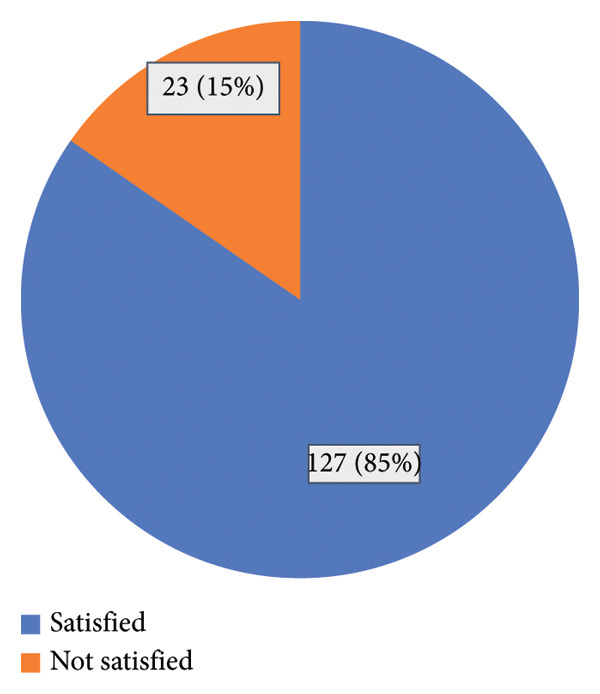
Satisfaction with nursing care among patients with CAD.

The mean PSNCQQ score (ranging from 19 to 95) was 56.48 ± 9.05. The study showed that 127 (85%) of the respondents were satisfied with nursing care (Figure 1).

### 3.3. The Distribution of Different Aspects of Patient Satisfaction Among the Respondents

About 60 (40%) of the respondents reported having given very good information about tests, treatments and what to expect, while 66 (44%) of respondents reported having very good instructions for preparing for tests and operations. About half, 76 (50.7%) of the participants reported having no difficulty getting information from the nurses willing to answer their questions, while 69 (46%) of respondents reported very good. Regarding the information given by nurses about communication with patients and their families, 73 (43%) of respondents replied good about family or friends’ involvement in their condition and needs. Also, 79 (52.7%) reported very good having involved family or friends in their care, and they were allowed to help. Most of the respondents 90 (60%) reported having good concern and caring shown by nurses on courtesy and respect they were given, friendship and kindness, while 85 (56.7%) of respondents reported having received very good attention by nurses to their condition and often checked and well‐kept track of how they were doing. Of the respondents, 66 (44%) reported having good recognition of their options and asked what they thought was important, and they were given choices.

About three‐quarters, 118 (78.7%) of respondents reported having given good consideration to their needs and the willingness of the nurses to be flexible in meeting their needs. Of the respondents, 110 (73.3%) reported having a very good daily routine of the nurses and how well they adjusted their schedules to their needs, while 99 (66%) of respondents reported very good helpfulness and the ability of the nurses to make them comfortable and reassure them. About 71 (47.3%) of respondents reported having very good skills and competence of nurses, and how well things were done, like giving medicine and handling intravenous insertions. Also, 79 (52.7%) of respondents reported very good coordination of care and teamwork between nurses and other hospital staff who took care of them. Of the respondents, 113 (75.3%) reported having a restful atmosphere provided by nurses, a sense of peace. Also, 90 (60%) of respondents reported having excellent provisions of privacy by nurses, while 97 (64.7%) reported having good discharge instructions, how clearly and completely the nurses told them what to do and what to expect when they left the hospital. In addition, 96 (64%) of respondents reported having a very good overall quality of care and services they received during the hospital stay (Table 2).

**Table 2:** Frequency and percentage distribution of responses per PSNCQQ item.

Variable	Response category	Frequency	Percent (%)
Information you were given: about tests, treatments and what to expect	Excellent	29	19.3
	Very good	60	40.0
	Good	38	25.3
	Fair	23	15.3

Instructions: to prepare for tests and operations	Excellent	24	16.0
	Very good	66	44.0
	Good	35	23.3
	Fair	25	16.7

Ease of getting information: willingness to answer your questions	Excellent	25	16.7
	Very good	76	50.7
	Good	38	25.3
	Fair	11	7.3

Information given by nurses: communicated with patients and families	Excellent	6	4.0
	Very good	69	46.0
	Good	69	46.0
	Fair	6	4.0

Informing family or friends about your condition and needs	Excellent	5	3.3
	Very good	54	36.0
	Good	73	48.7
	Fair	18	12.0

Involving family or friends in your care: how much they were allowed to help in your care	Excellent	23	15.3
	Very good	79	52.7
	Good	41	27.3
	Fair	7	4.7

Concern and caring by nurses: courtesy and respect, you were given; friendliness and kindness	Excellent	50	33.3
	Very good	90	60.0
	Good	9	6.0
	Fair	1	7

The attention of nurses to your condition: how often nurses checked on you and how well they kept track of how you were doing	Excellent	43	28.7
	Very good	85	56.7
	Fair	1	7

Recognition of your opinions: how much nurses ask you what you think is important and give you choices	Excellent	38	25.3
	Very good	40	26.7
	Good	66	44.0
	Fair	6	4.0

Consideration of your needs: the willingness of the nurses to be flexible in meeting your needs	Excellent	17	11.3
	Very good	118	78.7
	Good	14	9.3
	Fair	1	7

The nurses’ daily routine: how well they adjusted their schedules to your needs	Excellent	27	18.0
	Very good	110	73.3
	Good	12	8.0
	Fair	1	7

Helpfulness: the ability of the nurses to make you comfortable and reassure you	Excellent	35	23.3
	Very good	99	66.0
	Good	15	10.0
	Fair	1	7

Skill and competence of nurses: how well things were done, like giving medicine and handling intravenous insertion	Excellent	25	16.7
	Very good	71	47.3
	Good	53	35.3
	Fair	1	1

Coordination of care: the teamwork between nurses and other hospital staff who took care of you	Excellent	26	17.3
	Very good	79	52.7
	Good	43	28.7
	Fair	2	1.3

The restful atmosphere provided by nurses: the amount of peace	Excellent	36	24.0
	Very good	113	75.3
	Good	1	7
	Fair	0	0

Privacy: provisions for your privacy by nurses	Excellent	90	60
	Very good	56	37.3
	Good	4	2.7
	Fair	0	0

Discharge instructions: how clearly and completely the nurses told you what to do and what to expect when you left the hospital	Excellent	16	10.7
	Very good	27	18.0
	Good	97	64.7
	Fair	10	6.7

Overall quality of care and services you received during your hospital stay	Excellent	29	19.3
	Very good	96	64.0
	Good	25	16.7
	Fair	0	0

### 3.4. Factors Associated With Satisfaction With Nursing Care Provided Among the Respondents

The higher education level and treatment category were significantly associated with satisfaction status. Patients with higher education levels showed a significantly reduced likelihood of dissatisfaction with nursing care compared to those who were illiterate (adjusted odds ratio [aOR] = 0.478, 95% CI: 0.094–0.632, *p* = 0.012). Similarly, the patient’s payment method was significant, with insured patients having an aOR of 0.432 (95% CI: 0.371–0.921, *p* = 0.043) and VIP patients having an aOR of 0.234 (95% CI: 0.074–0.432, *p* = 0.002), both showing significantly reduced risk of dissatisfaction compared to those receiving out‐of‐pocket methods. Other variables included in the analysis, such as age, sex, marital status, occupation and education levels, did not show significant associations with satisfaction with nursing care (Table 3).

**Table 3:** Factors associated with satisfaction level with nursing care (*N* = 150).

Variable	Category	Univariate				Multivariate			
		COR	95% CI		*p* value	AOR	95% CI		*p* value
			Lower	Upper			Lower	Upper	
Age (in years)	18–44	Ref							
	45–64	2.875	0.609	13.569	0.182				
	65+	1.568	0.293	8.396	0.599				

Sex	Male	Ref							
	Female	0.459	0.177	1.191	0.109				

Marital status	Single	Ref							
	Widow/widower	0.542	0.083	3.514	0.520				
	Married	0.454	0.129	1.605	0.220				
	Divorced/separated	1.444	0.284	7.341	0.658				

Occupation	Employed	Ref							
	Peasant	3.619	0.452	28.990	0.226				
	Business	1.778	0.170	18.569	0.631				

Education level,	Illiterate	Ref				Ref			
	Primary	1.333	0.144	12.340	0.800	1.734	0.345	13.45	0.772
	Secondary	1.149	0.123	10.727	0.903	1.231	0.467	11.934	0.876
	Higher education	0.621	0.055	0.435	0.008	0.478	0.094	0.632	0.012

Patient’s payment method	Out‐of‐pocket	Ref				Ref			
	Insured	0.506	0.260	0.815	0.024	0.432	0.371	0.921	0.043
	VIP	0.114	0.129	0.742	0.001	0.234	0.074	0.432	0.002

Abbreviations: AOR = adjusted odds ratio, CI = confidence interval, COR = crude odds ratio, VIP = very important person.

## 4. Discussion

The study aimed to assess the level of satisfaction with nursing care and its associated factors among patients with CAD. The results of this study revealed a high prevalence of satisfaction among patients. The study revealed that respondents with health insurance were more likely to be satisfied with nursing care compared to those who had no insurance. Furthermore, having higher education was associated with being less likely to be satisfied with nursing care. Sociodemographic characteristics such as age, sex, occupation and marital status were not associated with satisfaction in nursing care.

The study revealed a high prevalence of satisfaction among patients with CAD regarding nursing care at JKCI, with 85% of respondents expressing satisfaction. The high level of satisfaction can be attributed to several key factors identified in the study, including information provision about tests and treatments, instructions for preparations and the overall clarity of discharge instructions. Additionally, the courtesy and respect shown by nurses, along with their attentiveness to patient conditions and involvement of family or friends in care, were consistently highlighted as positive aspects of nursing care. These findings align with a study reported in Saudi Arabia that emphasised the importance of effective communication and patient‐centred care in improving patient satisfaction [33]. However, our findings are based on patients only, while that in Saudi Arabia used both nurses and patients. Further, the findings in the current study are consistent with those reported in India, which elaborated the critical role of effective communication, empathy and patient‐centred care in enhancing satisfaction levels among healthcare recipients [18]. The high prevalence of satisfaction among patients with CAD regarding nursing care has significant implications for healthcare practices and policies. First, it underscores the importance of effective communication, empathy and patient‐centred care in improving patient satisfaction. Hospitals and healthcare providers can use these findings to prioritise and invest in training programs that enhance nurses’ communication skills and emotional intelligence. Additionally, the involvement of family and friends in patient care can be recognised as a critical component of holistic care strategies, suggesting that policies should encourage family participation where appropriate [19]. The results also indicate that clear and thorough information provision, including discharge instructions, plays a crucial role in patient satisfaction. Hence, healthcare institutions should ensure that their staff are well‐trained to deliver information effectively and consistently.

Demographic factors such as age, gender, marital status and occupation did not significantly influence satisfaction levels in this study. This finding is consistent with the quantitative study in East Asia, which found that sociodemographic factors did not significantly affect patient satisfaction when high‐quality, patient‐centred care was provided [34], while some previous research suggested that age was a significant predictor [18, 35]. The difference might be attributed to the fact that JKCI is a specialised cardiac centre and offers comprehensive cardiac services.

Respondents in the public category were more likely to be dissatisfied with nursing care than those with insurance and VIP. This finding is supported by the systematic review, which identified that financial barriers and the associated stress significantly impact patient satisfaction with healthcare services [36]. This is attributed to the high cost of health services at the institution because it is the national referral hospital for cardiac problems. This renders the cost and nursing services higher compared to other peripheral hospitals. This emphasises the universal effectiveness of the care practices at JKCI and supports the idea that high‐quality, patient‐centred care can transcend demographic differences.

However, the disparity in satisfaction between public category patients and those with insurance or VIP status highlights significant inequities in healthcare access and affordability. The higher dissatisfaction among public patients points to the financial barriers faced by this group, which can impede access to timely and necessary care. This underscores the need for policy interventions to address cost‐related issues and ensure equitable access to high‐quality cardiac care for all patients, regardless of their financial status.

Illiterate respondents had an increased risk of dissatisfaction compared to those with higher education. This finding aligns with that reported in Pakistan, which described the impact of educational level on patient satisfaction and the necessity of tailored communication strategies for patients with varying literacy levels [35]. This implies education is an important factor in understanding health services provided and their rationale because education increases the ability to understand the information provided in treatment. To address this, healthcare providers should implement health literacy programs, simplify communication and develop accessible educational materials. Training for nurses and staff on effective communication techniques for patients with low literacy levels is essential. Establishing support systems, such as patient navigators, and engaging in community outreach can further bridge the educational gap. Policy initiatives should mandate educational support to improve health equity, ensuring that all patients, regardless of literacy level, can understand and benefit from their healthcare, ultimately leading to higher satisfaction and better health outcomes.

Addressing the factors that contribute to high satisfaction, such as improving information dissemination, fostering respectful patient interactions and involving family members in care decisions, can enhance overall patient experiences and outcomes. Moreover, understanding the nuances of insignificant demographic factors helps healthcare providers tailor interventions more effectively, focusing on enhancing care quality rather than demographic profiling alone.

### 4.1. Limitation

This study is limited by the fact that it was conducted in a single specialised national cardiac centre, which offers comprehensive cardiac services. These findings, therefore, should be interpreted with caution, particularly when considering their applicability on a nationwide scale. Also, the connotation of ‘satisfaction’ is the gap between the patient’s expectation and the experience and most often is subjective. Thus, the validity and the usefulness of satisfaction data are limited.

## 5. Conclusion

Overall, patients expressed general satisfaction with the inpatient nursing care they received, and their perceived needs and care expectations from nurses significantly influenced their satisfaction levels. The key factors contributing to patient satisfaction included the professionalism and expertise of the medical staff, the quality of facilities and equipment and the comprehensive nature of treatment programs. Enhancing patients’ education concerning use of health insurance and education on CAD management underscores the importance of continuous evaluation to maintain and improve patient satisfaction.

## Consent

The authors have nothing to report.

## Disclosure

All authors reviewed and approved the final manuscript for publication.

## Conflicts of Interest

The authors declare no conflicts of interest.

## Author Contributions

Theresia I. Marombe created the study’s concept, created the methodology, gathered, examined, analysed, and interpreted data and penned the manuscript’s initial draft. Masunga K. Iseselo reviewed and edited the manuscript for important intellectual content. Samweli Kisakeni reviewed and edited by giving comments.

## Funding

The author(s) received no financial support for the research, authorship and/or publication of this article.

## Data Availability

The data that support the findings of this study are available from the corresponding author upon reasonable request.
